# A comparison of the polytomous Rasch analysis output of RUMM2030 and R (ltm/eRm/TAM/lordif)

**DOI:** 10.1186/s12874-019-0680-5

**Published:** 2019-02-20

**Authors:** Michael Robinson, Andrew M. Johnson, David M. Walton, Joy C. MacDermid

**Affiliations:** 1 Faculty of Health Sciences, The University of Western Ontario, London, ON Canada; 2 School of Health Studies, The University of Western Ontario, London, ON Canada; 3 School of Physical Therapy, The University of Western Ontario, London, ON Canada; 4 The University of Western Ontario, London, ON Canada; 5 Clinical Research Lab, Hand and Upper Limb Centre, St. Joseph’s Health Centre, London, Ontario Canada; 6 Science McMaster University, Hamilton, ON Canada

**Keywords:** Rasch, RUMM2030, R, DIF, IRT

## Abstract

**Background:**

Patient-reported outcome measures developed using Classical Test Theory are commonly comprised of ordinal level items on a Likert response scale are problematic as they do not permit the results to be compared between patients. Rasch analysis provides a solution to overcome this by evaluating the measurement characteristics of the rating scales using probability estimates. This is typically achieved using commercial software dedicated to Rasch analysis however, it is possible to conduct this analysis using non-specific open source software such a R.

**Methods:**

Rasch analysis was conducted using the most commonly used commercial software package, RUMM 2030, and R, using four open-source packages, with a common data set (6-month post-injury PRWE Questionnaire responses) to evaluate the statistical results for consistency. The analysis plan followed recommendations used in a similar study supported by the software package’s instructions in order to obtain category thresholds, item and person fit statistics, measures of reliability and evaluate the data for construct validity, differential item functioning, local dependency and unidimensionality of the items.

**Results:**

There was substantial agreement between RUMM2030 and R with regards for most of the results, however there are some small discrepancies between the output of the two programs.

**Conclusions:**

While the differences in output between RUMM2030 and R can easily be explained by comparing the underlying statistical approaches taken by each program, there is disagreement on critical statistical decisions made by each program. This disagreement however should not be an issue as Rasch analysis requires users to apply their own subjective analysis. While researchers might expect that Rasch performed on a large sample would be a stable, two authors who complete Rasch analysis of the PRWE found somewhat dissimilar findings. So, while some variations in results may be due to samples, this paper adds that some variation in findings may be software dependent.

## Background

Patient-reported outcome measures (PRO) have traditionally been developed using Classical Test Theory (CTT) [1]. PRO are commonly comprised of ordinal level items on a Likert response scale and suffer from having an inconsistent or unknown difference between the levels on the scale [1]. This inconsistency makes rating scales that use ordinal level (Likert-style) items problematic when trying to compare results between patients [1], and violate the assumptions of most statistical tests.

Rasch analysis is a modern measurement method that overcomes some of these limitations in classical approaches. Rasch analysis is a probabilistic model that uses an analytical model developed by Danish mathematician George Rasch, called the Rasch model. The Rasch model can be used to evaluate the measurement characteristics of rating scales using probability estimates [2]. Specialized statistical software packages have been developed that are dedicated to Rasch analysis, and that do not require the end user to develop custom statistical functions to fit the models. Conversely, open-source software does not provide this level of user-friendliness but is more accessible; and has a platform on which to develop code that can conduct Rasch analyses. Technical comparisons of dedicated software versus open-source coding can inform our understanding of analytical approaches. While there are a few software packages available, this paper will evaluate the most commonly used commercial package (RUMM 2030) against four free, open-source packages available for R (an open-source statistical programming language) to determine if there is consistency among the results [3–5]. It is useful to understand whether different analytical plans would provide different results. The Patient-rated Wrist Evaluation is an ideal choice for such an analytical comparison since it has already been subject to multiple Rasch analyses [6–8].

### The Rasch model

The Rasch model was developed by George Rasch and is a method of testing a rating scale against a mathematical measurement model that assumes person-level responses to an individual item estimate their actual position on the continuum of the latent construct, and that their position on the latent construct should be estimable only by their responses to each individual item [2, 9]. The Rasch model separates persons by their location on the theoretical continuum of the underlying construct by locating the response thresholds between adjacent response options for each item along a logit based continuum. The scale is tested against the Rasch model using the logit based location, and once the scale fits the model, the position of the response thresholds is transformed into an interval scale [2, 9].

Rasch analysis begins by ordering all possible response options to all items and all persons along a unitless logit-transformed continuum representing the levels of the latent construct (from very low to very high). It then statistically evaluates the hypothesis that people located higher on the continuum should show a higher likelihood of choosing response options that are also located higher on that same continuum [2]. The statistical calculations employed by the Rasch model to locate and order persons and item difficulty is based on Guttmann Scaling [10]. Guttmann scaling is a deterministic pattern with a strict hierarchical ordering of items that assumes “agreement” with all items of lower rank when a particular item is affirmed [2, 10]. Rating scales that are evaluated against the Rasch model can then be evaluated for the psychometric properties of consistency, reliability and responsiveness [2, 10, 11]. Rasch analysis can be applied to a variety of situations including the development of new rating scales, the analysis of the psychometric properties of existing scales, during hypothesis testing of the structure of ordinal scales, for constructing item banks and for calculating change scores from ordinal scales [10].

Rasch analysis can be used with both dichotomous and polytomous data sets via the dichotomous model or either of the polytomous models (Andrich Rating Scale Model and Masters Partial Credit Model) [9, 10]. The two polytomous models use the same Rasch model but the Andrich Rating Scale Model expects there to be an equal difference between item thresholds [9, 10].

### Fit statistics

The Rasch model takes three different types of fit statistics into consideration, two item-person interaction statistics and one item-trait interaction statistic [10, 12]. The item-person interaction statistics provide a summary of all the item or person deviations from the Rasch model by standardizing the individual item and person fit residuals (the difference between the observed score and expected score) to approximate a Z-Score, where Z-scores between ±2.5 indicate an adequate fit to the model [10, 12, 13]. Item fit can be represented graphically by plotting the responses for each of the class intervals against the Rasch model’s item characteristic curve [12]. Two chi-square ratios, infit and outfit mean square statistics, are used to determine how well the data meets the requirement of the Rasch model [13]. The chi-square values are divided by their degrees of freedom in order to establish a ratio scale with an expected value of + 1 and can range from 0 to infinity [13]. For the item-trait interactions chi-square values for each of the individual items are obtained, combined then evaluated for statistical significance using the summed degrees of freedom [10, 12]. The chi-square statistics should indicate a non-significant deviation from the Rasch model after adjustment for multiple tests [10, 12].

### Unidimensionality

Unidimensionality refers to the ability of the rating scale to focus on and measure one attribute at a time [2, 13]. RUMM2030 uses Principal Component Analysis (PCA) to detect any signs of multidimensionality by evaluating the residuals for meaningful patterns; if these patterns are absent, this is taken to indicate unidimensionality [5, 14]. An alternative method to evaluate unidimensionality is via a conditional likelihood ratio test developed by Martin-Löf [15]. This test, called the Martin-Löf-Test, tests whether unidimensionality holds for different subgroups of items [14, 16, 17].

### Category thresholds

The category thresholds of rating scales are the point at which a person is equally likely to select two adjacent response options [18, 19]. The examination of category thresholds involves the inspection of category probability curves to determine if the response probabilities are arranged in ascending order concordant with the categories, which would indicated ordered thresholds. If the response probabilities are arranged in reverse order, this would indicate disordered thresholds [18–20]. Too many options or poor category definition are two sources of disordered categories which can cause item misfit as a result of inconsistent responses from patients [18, 19]. When category thresholds are identified as being disordered, the problem is frequently due to having too many response options, and this can usually be resolved by collapsing responses, as long as some general guidelines are taken into account [18, 19]. The resulting collapsed category thresholds must be logical and make sense, and there should be an attempt to create a uniform frequency distribution across the new categories [13]. The reliability and validity indicators of resulting category thresholds should then be assessed in order to evaluate how the new rating scale is functioning overall [13].

### Differential item functioning and item bias

Differential item functioning (DIF), also referred to as item bias, occurs when different groups possess comparable levels of the trait being measured but respond differently to the individual items [10, 21, 22]. There are two types of DIF that Rasch analysis identifies, uniform DIF and nonuniform DIF [9]. Uniform DIF occurs when the group displays a consistent difference in their responses whereas nonuniform DIF occurs when the group displays inconsistent differences in their responses [9, 10, 21]. Uniform DIF can be resolved by splitting items into the different person factor groups where the DIF was identified. Alternatively, the items with DIF can be grouped together in a subtest to determine if the DIF cancels out at the test level [5]. Non-uniform DIF requires the removal of the particular item [10, 21]. If any of these procedures are carried out then the remaining items should be retested to determine if this has affected the scale or results in issues with statistical power [5, 9, 10, 18].

### Local independence

Local independence is an assumption of the Rasch model and can be evaluated through response dependency and multidimensionality [9]. Response dependency occurs when items are linked in such a manner that sees the response to one item determining the response to another item [5, 9, 10]. The relationship between the underlying construct for each item can be identified by inspecting the residual correlation matrix, and correlations less than 0.28 are generally considered to be acceptable however, a new simulation study suggests that correlations less than 0.2 above the average be adopted instead [10, 23, 24]. As the number of items has a direct influence on the average, one must take into consideration that the residual correlations are relative to the overall set of correlations [14]. When a violation of this assumption occurs, items may have to be removed, or correlating items may have to be grouped together, in order to help improve the model fit [10, 23].

### Person separation index

One additional measurement outcome that can be obtained is the Person Separation Index (PSI) which is interpreted in the same way as Cronbach’s alpha [9, 10] In fact, the only calculation difference between PSI and Cronbach’s alpha lies within the value used within the formula, with PSI using the logit value and Cronbach’s alpha using the raw value [9]. The PSI is an indication of reliability and reflects the ability to differentiate between different levels of the underlying construct [9, 10].

## Software

### RUMM2030

RUMM2030 (2012) is a statistical software package developed by the Perth Australia based RUMM Laboratory Pty Ltd. [5] This software package is Windows based and provides a graphical user interface for conducting Rasch analysis. As RUMM2030 is a commercial product there is a licensing fee, which varies depending on the edition purchased [5]. Both editions of RUMM2030 provide basic tools in order to conduct Rasch analysis; however, the professional version provides standard errors for thresholds, provides strategies for examining item linked response dependencies, allows for facet analysis of 3-way response structures, provides conditional tests-of-fit for pairs of polytomous items or tests, allows for tailored post-hoc response analysis and more advanced graphical output (enhanced threshold maps, Person Characteristic Curves and standard residual plots) [5].

### R

R is a language designed to provide a framework for statistical analysis and graphical representations of data [25–27]. R was originally developed by John Chambers at the former Bell Laboratories and is licensed under the Free Software Foundation’s GNU General Public License [25–27]. Natively, R provides a command line environment to handle and store data, perform calculations and also includes a core collection of tools for data analysis [25–27] and R also provides the flexibility for third-party developers to build custom scripts to implement specific analyses. In order to make R more user friendly, there are a variety of third party programs that provide a user friendly interface for package management, file importation and more features (e.g. R Studio, R Commander) [25–27].

### Patient-rated wrist evaluation

The Patient-Rated Wrist Evaluation is a 15-item, patient-reported questionnaire that was developed to assess wrist joint pain and functional difficulties following an injury to the wrist joint and surrounding area [28, 29]. It is considered a core outcome for distal radius fracture [30, 31]. The PRWE was rigorously developed and found to be a reliable and valid measure of patient-rated wrist pain and disability [32, 33]. The PRWE consists of 15 items separated into two subscales: Pain Subscale (5 items with responses ranging from 0 = no pain to 10 = worst pain ever) and the Function Subscale (10 items ranging from 0 = no difficulty to 10 = unable to do) [28]. The Function Subscale is further divided into Specific Activities (6 items) and Usual Activities (4 items) [28].

## Methods

### Participants

This study used a cross-sectional data set consisting of the 6-month post-injury PRWE scores of 382 (88 males, 293 females, mean age 57 ± 13.5) patients recruited from Roth| McFarlane Hand and Upper Limb Centre in London, Ontario, Canada. The patients were all 18 years of age or older; patients who could not read and write English, had a cognitive impairment, or who were unable to provide consent or complete the PRWE, were excluded. Informed consent was obtained from all participants prior to being admitted into the original study that formed the data set. Medically unstable patients or those with a life-threatening comorbidity were also excluded. The sample included 346 right handed and 36 left handed individuals.

### Analysis plan

The analysis plan followed the same recommendations used by a similar study for the examination of polytomous rating scales using Rasch analysis [10, 11]. The specific procedures for each software package were followed as outlined by each developer [3–5, 34, 35]. The PRWE data set imported into RUMM2030 version 5.4 (RUMM Laboratory Pty Ltd., Perth, Australia) and R Studio version 1.0.136 (R Studio Inc., Boston, MA). The analysis was then performed using RUMM2030 and four packages within R: ltm version 1.0–0, eRm version 0.15–7, lordif version 0.3–3 and TAM version 1.99999–31. Once the Rasch analysis was performed, the output from each software package was then compared to determine if there was consistency within the results.

The objective of the analysis plan is to subject the same PRWE data set to Rasch analysis using RUMM2030 and R to be able to compare the analytic approaches and output. To accomplish this the PRWE was evaluated for construct validity by using Rasch analysis to evaluate the unidimensionality and reliability of the 3 subscales, for fit to the Rasch model by examining the interval properties and ordering of item thresholds of the 3 subscales and if there was an age or sex-linked item bias within the 3 subscales.

The following steps were completed to obtain the output from RUMM2030:

1. To determine the appropriate Rasch model to use, a log-likelihood ratio test was performed. The purpose of the log-likelihood ratio test is to take the unrestricted parameterization of the model (i.e. no contains were placed on the items parameters) and assess it against the rating re-parameterization of the same model [5]. A non-statistically significant result indicates that the rating scale model should be used, whereas a statistically significant result indicates that the partial credit model should be used instead [10].
2. Category probability plots were constructed to establish the category thresholds for the rating scale. The re-scoring of disordered thresholds were corrected by collapsing categories then re-constructing the probability plots to ensure that the disordered thresholds were eliminated [10].
3. Item fit was evaluated by analyzing the item fit residual statistics and an item-trait interaction Chi-Square statistic [10]. Item fit z-score transformed residuals between ±2.5 are deemed to indicate adequate fit to the model [10].
4. Person fit was evaluated by using the same procedure as above for item fit.
5. The Person Separation Index (PSI) is a measure of reliability and is interpreted in the same way as Cronbach’s alpha [2, 10, 36]. The PSI determines the number of distinct subgroups within the data set, the number of comparative groups exist within the data set and if the rating scale is sufficiently robust to allow for group or individual comparisons [10, 36].
6. Differential Item Functioning (DIF) was then evaluated to determine if different groups of respondents, who possessed equal levels of the trait being measured, responded differently to the question [2, 10, 37]. DIF was evaluated by examining the item residuals statistically with a between groups analysis of variance (ANOVA), and graphically by plotting item characteristic curves (ICC) for age, sex, diagnosis and hand dominance [9, 10, 37].
7. To check for local dependency within the items, an analysis of the correlation of item residuals was performed [10]. This analysis looked for correlations > 0.3 which identified response linked items [10].
8. The unidimensionality of the subscales was analyzed in order to verify that each scale was only measuring one underlying construct [2, 10, 38]. Factor analysis was performed to evaluate principle component item loadings and then paired t-tests were conducted using the positively and negatively loaded items [2, 10, 38]. Unidimensionality is present if the percentage of significant t-test (at *P* < 0.05) is less than 5% [2, 9, 10, 38, 39].

The following steps were completed to obtain the output from R:

1. To determine the appropriate Rasch model to use, a log-likelihood ratio test was performed using the ltm package [34]. A non-statistically significant result indicates that the rating scale model should be used, whereas a statistically significant result indicates that the partial credit model should be used instead [10].
2. Category probability plots were constructed to establish the category thresholds for the rating scale using the TAM and eRm packages [35, 40]. The re-scoring of disordered thresholds were corrected by collapsing categories, then re-constructing the probability plots to ensure that the disordered thresholds were eliminated [10].
3. Item fit was evaluated by analyzing the item fit residual statistics using the TAM package [40]. Item fit residuals between ±2.5 are deemed to indicate adequate fit to the model [10].
4. Person fit was evaluated by using the same procedure as above for item fit.
5. As the PSI has not been implemented in an R package, Cronbach’s Alpha was obtained using the ltm package instead [2, 10, 34, 36].
6. The package lordif will be used to evaluate for DIF [41]. In order to detect the type of DIF, lordif uses a likelihood ratio *X*^2^ test to compare the nested models (Model 1: explanatory variable; Model 2: explanatory variable + vector of group identifiers; Model 3: explanatory variable x vector of group identifiers). A significant result between Model 1 and Model 2 would indicate the presence of uniform DIF and a significant result between Model 2 and Model 3 would indicate the presence of non-uniform DIF [41].
7. To check for local dependency within the items, an analysis of the correlation of item residuals was performed using ltm [10, 34]. This analysis looked for correlations > 0.3 above the average residual correlation which identifies response linked items [7, 10].
8. The unidimensionality of the subscales was analyzed in order to verify that each scale was only measuring one underlying construct [2, 10, 38]. Using the eRm package the Martin-Loef-Test was used and a statistically significant result represents a violation of unidimensionality [16, 35].

## Results

**Fig. 1:**
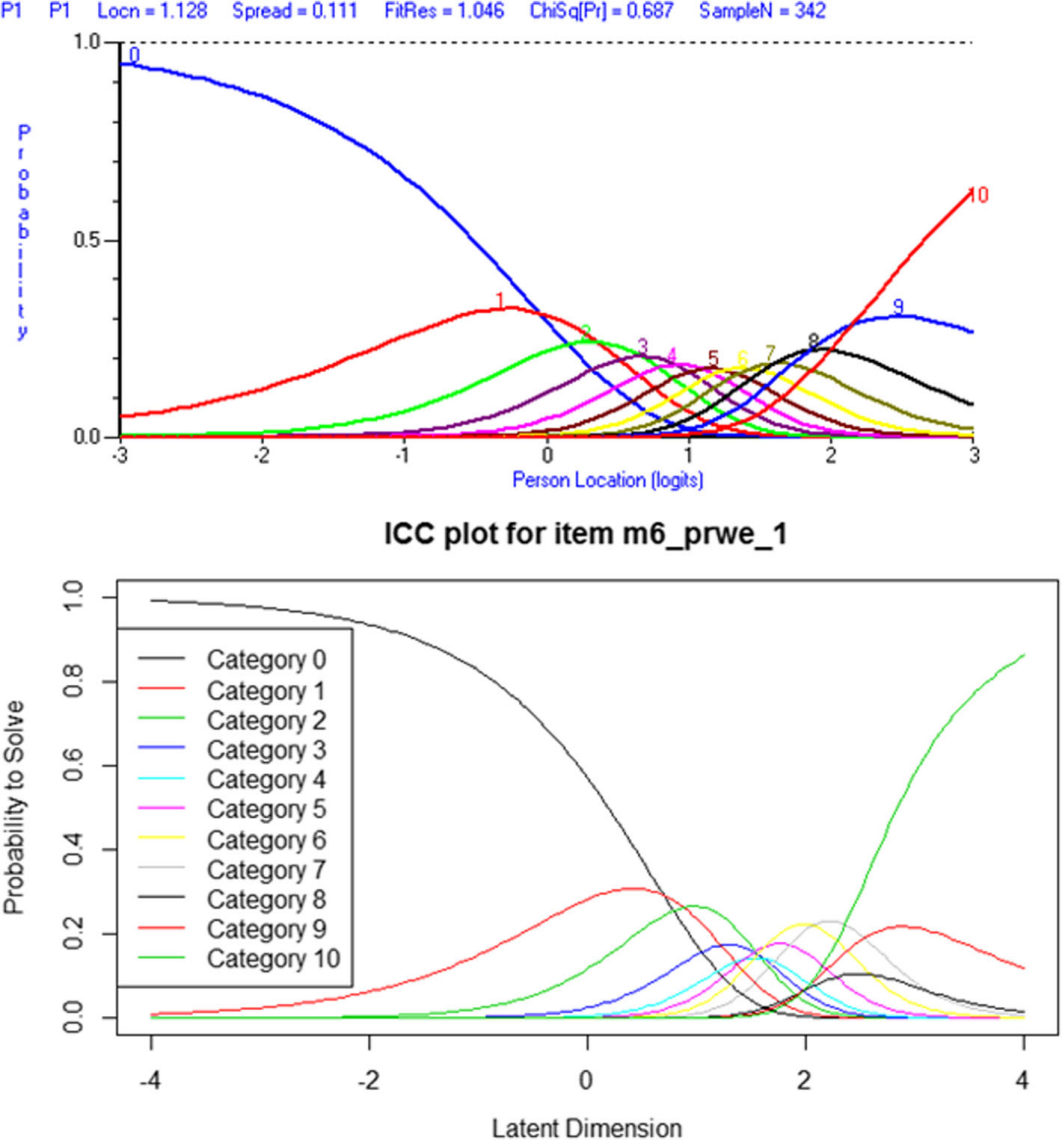
Example Item Characteristic Curve

**Fig. 2:**
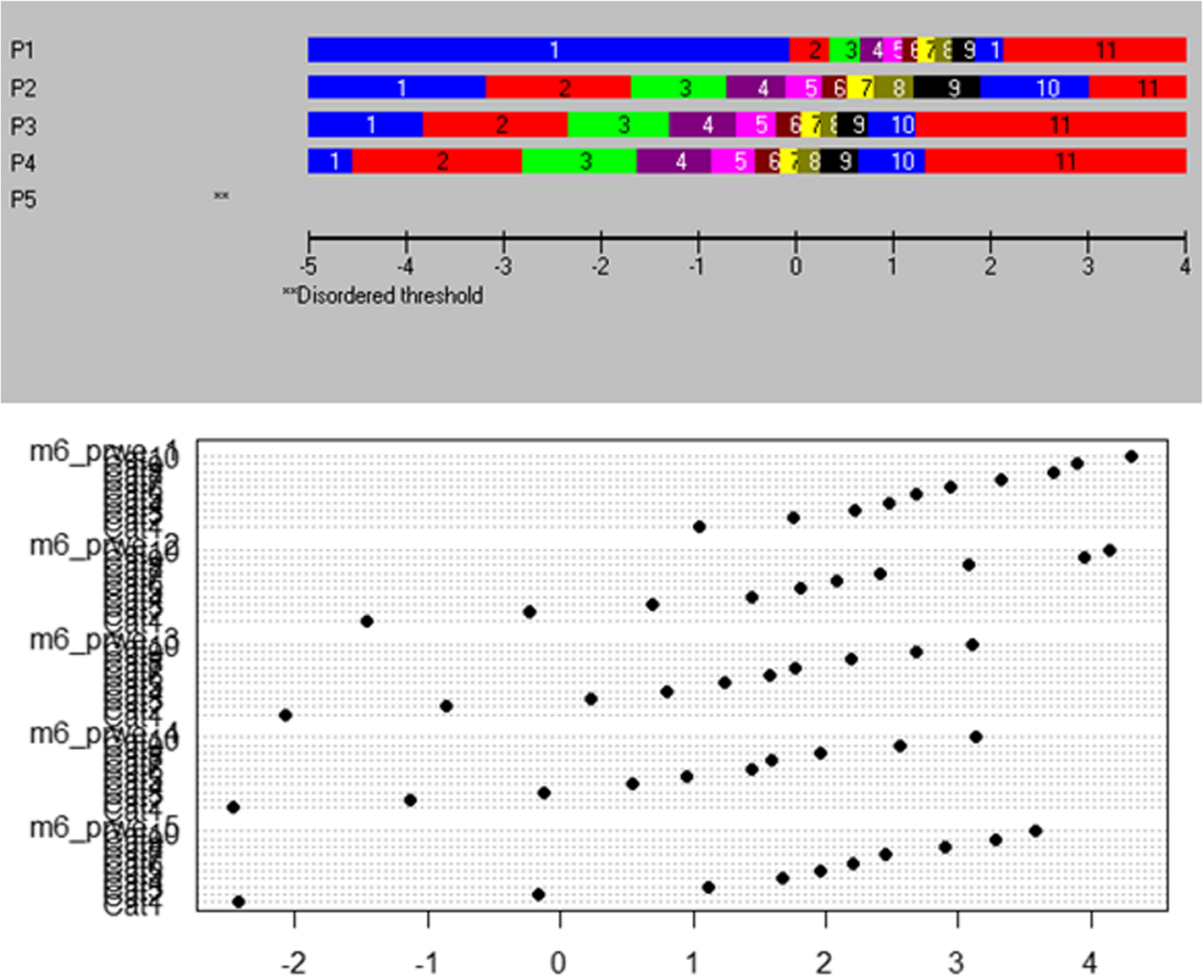
Example Threshold Map

**Fig. 3:**
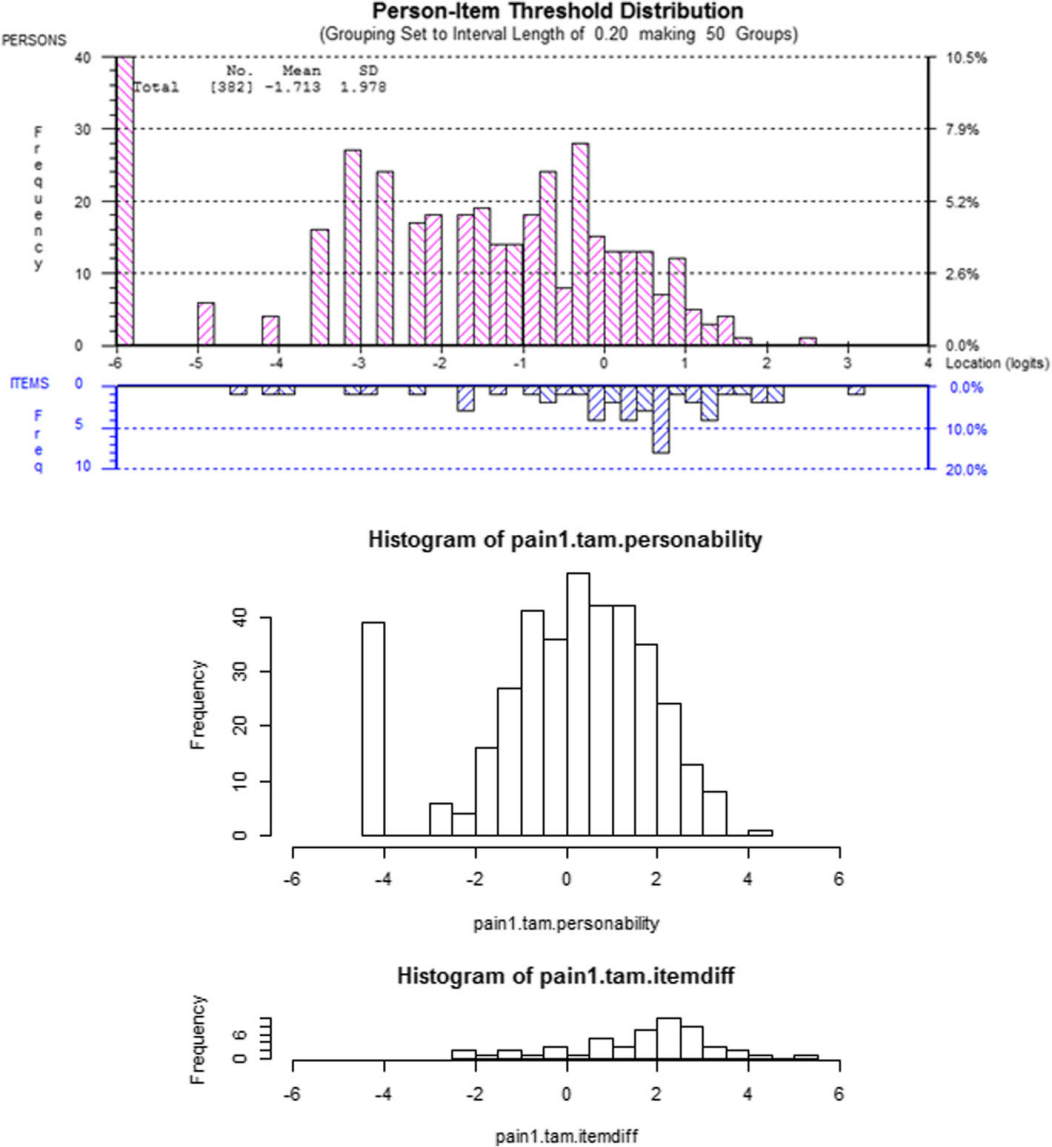
Example Person-Item Threshold Distribution Chart

The results of the Rasch analysis carried out on the 3 subscales is presented in Table 1 and the category threshold locations are presented in Tables 2, 3, 4, 5. Apart from the final results for the usual subscale and the evaluation of the category thresholds, there is consistency within the final outcomes from both software packages; however, there are significant inconsistencies between the output of the software packages. Example item characteristic curves, threshold maps and person-item threshold distribution charts are presented in Figs. 1, 2 and 3 respectively. There does not appear to be a substantively significant amount of discrepancy between the graphical output from the two software packages; however, RUMM2030 centralizes the mean location of items at 0 which does alter the magnitude of scales used in the graphs [5].

**Table 1:** RUMM2030 and R Output

			Item Fit Residual^a^		Person Fit Residual^a^		Chi-Square^b^		PSI^c^		UNID T-Test^d^	
			Mean	SD	Mean	SD	Value (DF)	p	With	without		
RUMM 2030	PAIN	Initial	−0.8398	1.7301	−0.4845	0.9770	24.391 (20)	0.4969	0.9218	0.9135	4.6800%	
		Final	−0.5336	1.1746	−0.4492	0.9127	18.5992 (20)	0.5480	0.8781	0.8644	2.3500%	
	SPECIFIC	Initial	−0.6052	2.1366	−0.4538	1.0702	54.0898 (30)	0.0045	0.9220	0.9033	4.7800%	
		Final	−0.6266	1.5595	−0.4497	0.9686	50.3341 (20)	0.0002	0.9016	0.8699	3.6800%	
	USUAL	Initial	−0.4119	1.7207	−0.4544	0.9432	23.1212 (20)	0.2829	0.9208	0.8304	6.1600%	
		Final	−0.3182	1.8380	−0.4642	1.0076	32.1662 (20)	0.0416	0.9217	0.8888	5.0700%	
			Item Fit Residual^a^		Person Fit Residual^a^				Cronbach’s Alpha^c^		Martin-Loef^d^	
			Mean	SD	Mean	SD					Value (DF)	p
R	PAIN	Initial	1.63322	1.6195	−0.0447	1.89779			0.922		370.171(599)	1.0000
		Final	1.7807	1.6219	0.0067	1.7558			0.872		239.851(339)	1.0000
	SPECIFIC	Initial	1.6092	0.80491	0.12174	1.37694			0.92		392.039(899)	1.0000
		Final	1.7810	1.0349	0.1273	1.3960			0.8980		246.659(399)	1.0000
	USUAL	Initial	2.73459	1.43031	0.27866	1.84451			0.921		216.992(399)	1.0000
		Final	2.9494	1.9276	0.2744	2.0210			0.9200		ERROR	n/a

^a^ The fit residuals should have mean of 0 ± 2.5 and a standard deviation of 1 ± 2.5

^b^ The Chi-Square statistic should be small and statistically non-significant

^c^ A Person Separation Index (PSI) or Cronbach’s Alpha should be > 0.70 to be statistically reliable

^d^ Unidimensionality is present if the percentage of statistically significant t-tests is < 5% or if the result of the Martin-Loef test is not significant

**Table 2:**
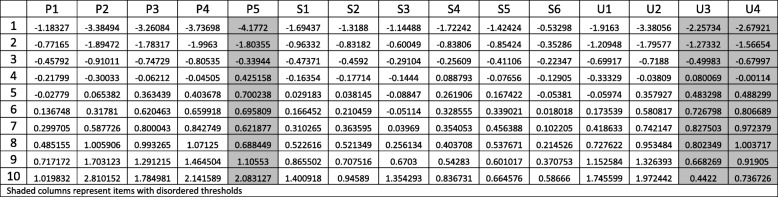
RUMM2030 Initial Category Thresholds

Shaded columns represent items with disordered thresholds

**Table 3:** RUMM2030 Final Category Thresholds

	P1	P2	P3	P5	S1	S2	S3	S5	U1	U2	U3	U4
1	−1.19835	−3.33737	−3.14927	−4.03637	−2.04266	−1.62154	−1.53629	−1.66087	−2.37087	−3.82445	−2.15655	−2.60126
2	−0.7825	−1.90999	−1.77635	− 1.6777	−1.18107	− 1.01629	−0.88369	−1.00577	− 1.64775	− 2.17004	−1.56105	− 1.72138
3	− 0.43429	− 0.93802	− 0.78829	−0.12234	− 0.63027	−0.57846	− 0.53659	−0.4986	− 1.03285	− 1.00179	− 0.50071	− 0.57784
4	−0.14711	− 0.30817	− 0.10941	0.825101	− 0.30001	− 0.26485	− 0.38711	− 0.11611	− 0.5064	− 0.2143	0.671801	0.638433
5	0.085634	0.092844	0.335983	1.359987	−0.10007	−0.03228	− 0.32736	0.16494	− 0.0486	0.297853	1.603822	1.736526
6	0.270551	0.378322	0.623587	1.6777	0.059794	0.162421	−0.24946	0.367776	0.36034	0.640062	1.942691	2.525522
7	0.414248	0.661548	0.829091	1.97362	0.269804	0.362451	−0.0455	0.515642	0.740206	0.917733		
8	0.523331	1.055814	1.028186		0.620198	0.610989	0.392384	0.631776	1.110787	1.236273		
9	0.604405	1.674409	1.296563		1.201208	0.951222	1.172094	0.739416	1.491874	1.701084		
10	0.664076	2.630623	1.709914		2.103069	1.426333	2.401514	0.861799	1.903255	2.417573

**Table 4:** R Initial Category Thresholds

	P1	P2	P3	P4	P5	S1	S2	S3	S4	S5	S6	U1	U2	U3	U4
1	1.043976	−1.4631	−2.06351	−2.46048	−2.418	−0.15353	0.04953	0.499969	−0.4747	−0.69315	0.535126	0.987213	−0.59079	−0.00357	− 0.66714
2	1.752594	−0.23245	−0.86288	−1.12564	− 0.1572	0.65799	0.674469	1.133881	0.491547	0.190338	0.981171	1.802582	0.73526	0.991608	0.494843
3	2.218781	0.691315	0.224579	−0.12167	1.110809	1.073822	1.072723	1.510346	0.902252	0.583649	1.249603	2.562836	1.716522	1.699677	1.420624
4	2.475494	1.441681	0.800079	0.548859	1.674957	1.419342	1.439301	1.750763	1.331451	0.90683	1.45816	3.006317	2.381927	2.395111	1.996307
5	2.678009	1.816132	1.242279	0.956085	1.955841	1.697479	1.655914	1.871979	1.534515	1.19577	1.617096	3.333527	2.862946	2.676178	2.444916
6	2.944427	2.079437	1.575897	1.440948	2.207245	1.933319	1.92746	2.069183	1.738312	1.462372	1.74472	3.777191	3.338287	3.088531	3.013641
7	3.320343	2.403534	1.772552	1.592743	2.455719	2.187103	2.045563	2.240936	1.855133	1.623322	1.891937	4.108978	3.63089	3.217438	3.203522
8	3.72226	3.080658	2.197906	1.957672	2.8992	2.467621	2.336884	2.581512	2.052155	1.821991	2.178131	4.219025	3.85556	3.386261	3.356781
9	3.895294	3.950409	2.684784	2.563202	3.275299	2.813507	2.69101	2.901581	2.394745	2.095001	2.525665	4.95401	4.28183	3.747162	3.627228
10	4.305267	4.138641	3.108856	3.136871	3.585114	2.933807	3.047333	3.067474	2.822845	2.536835	2.853241	5.552765	5.144806	3.918182	3.953888

**Table 5:** R Final Category Thresholds

	P1	P2	P3	P5	S1	S2	S3	S5	U1	U2	U3	U4
1	0.99527	−1.38327	−1.91666	−2.24918	−0.26633	−0.05649	0.417572	−0.8226	0.950043	−0.70541	−0.09256	−0.78214
2	1.698029	−0.24912	−0.8334	−0.18796	0.583099	0.599579	1.100006	0.085602	1.839752	0.68454	0.94162	0.411896
3	2.177033	0.633453	0.179718	1.025665	1.035919	1.033722	1.533051	0.500519	2.749237	1.748383	1.694	1.388031
4	2.44162	1.379242	0.735809	2.033112	1.428131	1.451569	1.824371	0.850433	3.335907	2.521088	3.007233	2.694489
5	2.648163	1.762115	1.175262	2.593414	1.758087	1.709564	1.976898	1.173981	3.810516	3.137238	4.294647	4.302521
6	2.915131	2.033295	1.514557	3.38736	2.051971	2.048676	2.23764	1.482513	4.50943	3.838715		
7	3.283722	2.365631	1.716705	3.653961	2.39035	2.201935	2.478607	1.673126	4.974701	4.290985		
8	3.673187	3.048065	2.155609		2.792816	2.601654	2.988922	1.913361	5.10434	4.617828		
9	3.840912	3.905731	2.652191		3.291412	3.095306	3.45163	2.249908	5.799042	5.150116		
10	4.239716	4.090668	3.078278		3.452911	3.559296	3.67099	2.784943	6.302765	6.009247

### Pain subscale

Only RUMM2030 detected disordered thresholds for item # 5 (How often do you have pain?), local dependency between item #1 (At rest) and item #5 (How often do you have pain?), and reached comparable conclusions regarding item fit, person fit, reliability and unidimensionality. Non-uniform DIF was identified by RUMM2030 and R for item #4 (When it is at its worst) for age. After removing item #4 and re-scoring item #5 (How often do you have pain?), the analysis was conducted a second time to re-evaluate the subscale and both programs reached similar conclusions (good item and person fit, a high reliability index and unidimensionality) and neither detected the presence of DIF.

### Specific activities subscale

The specific activities subscale did not demonstrate any issues with regards to threshold ordering using either RUMM2030 or R. Both RUMM2030 and R determined that the subscale displayed acceptable item and person fit statistics, good reliability and unidimensionality. RUMM2030 was able to determine that there was local dependence between item #1 (Turn a door knob using my affected hand) and item #2 (Cut meat using a knife in my affected hand); however, this conclusion was not reached using the output from R. RUMM2030 and R detected the presence of uniform DIF for item #4 (Use my affected hand to push up from a chair) for age, however only R detected uniform DIF for item #6 (Use bathroom tissue with my affected hand) for sex. After removing item #4 4 (Use my affected hand to push up from a chair) and item #6 (Use bathroom tissue with my affected hand), neither RUMM2030 or R detected the presence of DIF, and both reached similar conclusions regarding item fit, person fit, reliability and unidimensionality.

### Usual activities subscale

The usual activities subscale displayed disordered thresholds for item #3 (Work) and item #4 (Recreational activities) only in RUMM2030. There was disagreement between RUMM2030 and R regarding the item fit statistics (RUMM2030 determined there to be acceptable item fit whereas R did not) and unidimensionality (RUMM2030 determined that the subscale was not unidimensional whereas R did); however, comparable conclusions regarding person fit, local dependency and reliability were present. RUMM2030 did not detect the presence of DIF however R did detect the presence of non-uniform DIF for item #2 (Household work) for sex. After re-scoring item#3 (Work) and #4 (Recreational activities) the subscale was re-analyzed in both software packages. RUMM2030 determined that the re-scored subscale now displayed good item fit, person fit, reliability and acceptable levels of unidimensionality; however, R still disagreed with regards to item fit, but determined comparable conclusions regarding person fit and reliability. The R-based Martin-Loef test for the re-scored subscale resulted in an error and was not obtained.

## Discussion

While there appears to be substantial agreement between RUMM2030 and R with regards for most of the results, there are some small discrepancies between the output of the two programs. Table 6 provides an overview of the capabilities of each software package and Table 7 provides a comparison on the conclusions reached by RUMM2030 and R. The author of the eRm package for R does note that each package will produce different results due to the methods employed by the packages [35]. Additionally, RUMM2030 automatically centralizes the item locations to 0 whereas ltm, eRm, lordif and TAM use uncentralized item locations [34, 35, 40]. With regards to category thresholds, RUMM2030 identified 3 items with disordered thresholds where R did not identify any disordered items. This disagreement is not unexpected however as RUMM2030 uses Rasch-Andrich thresholds while R-TAM uses Thurstonian thresholds. With regards to differential item functioning, RUMM2030 only detected DIF for 2 items whereas R detected DIF for 4 items. There was agreement between the programs for the 2 items that was identified by RUMM2030. This difference could be explained by the difference in approaches taken by the programs (RUMM2030 examined the item residuals with a between groups ANOVA where R uses a likelihood ratio *X*^2^ test to compare the models) [41].

**Table 6:** Comparison of the Capabilities of each Software Package

	RUMM2030	R-ltm	R-eRm	R-TAM	R-lordif
Model Fit Statistics	YES	YES	YES	YES	NO
Unidimensionality	YES	YES^a^	YES^d^	YES	NO
Category Thresholds	YES	NO	YES	YES	YES
DIF	YES	YES^a^	YES^a^	YES	YES
Local Independence	YES	YES	YES	YES	NO
Reliability	YES^b^	YES^c^	YES^e^	YES^f^	NO

^a^ Dichotomous Data Only

^b^ Person Separation Index

^c^ Cronbach’s Alpha

^d^ Martin-Loef-Test

^e^ Conditional maximum likelihood framework

^g^ EAP and WLE reliability statistics

^g^ Joint maximum likelihood

**Table 7:** Comparison of the Results Obtained from RUMM2030 and R ltm/eRm/TAM/lordif

		RUMM2030	R- ltm/eRm/TAM/lordif
Model Fit Statistics	Pain	Acceptable Item and Person Fit	Acceptable Item and Person Fit
	Specific Activities	Acceptable Item and Person Fit	Acceptable Item and Person Fit
	Usual Activities	Acceptable Item and Person Fit	Acceptable Item Fit Unacceptable Person Fit
Unidimensionality	Pain	Unidimensionality Present	Unidimensionality Present
	Specific Activities	Unidimensionality Present	Unidimensionality Present
	Usual Activities	Unidimensionality Not Present	Unidimensionality Present
Category Thresholds	Pain	Disordered - Item #5	Ordered
	Specific Activities	Ordered	Ordered
	Usual Activities	Disordered - Items #3 & 4	Ordered
Differential Item Functioning	Pain	Non-uniform DIF – Item #4 for age	Non-uniform DIF – Item #4 for age
	Specific Activities	Uniform DIF – Item #4 for age	Uniform DIF – Item #4 for age, item #6 for sex
	Usual Activities	None Detected	Non-uniform DIF – Item #2 for sex
Local Independence	Pain	Local Dependency - Items #1 & 5	No Local Dependency
	Specific Activities	Local Dependency - Items #1 & 2	No Local Dependency
	Usual Activities	No Local Dependency	No Local Dependency

Aside from the underlying statistical approaches that are implemented (e.g., Person Separation Index vs. Cronbach’s Alpha) there are marked differences between the graphical output of each program. RUMM2030 is a purpose-built program that does not afford itself to customizations by the end user. While this does provide attractive, easy to read graphs and charts, it does not have the flexibility that R affords. R does allow for customization of the graphical output by the end user; however, the default output can be hard to read and interpret (see Fig. 1), and customization requires a working knowledge of other R packages. There is a substitutional trade off in this regard, as RUMM2030 provides a more user-friendly method of generating graphical output, where R provides a complex, non-user-friendly method of generating graphical output. Apart from the attractiveness of the output, both programs do produce comparable item characteristic curves and person-item threshold distribution maps. There is a marked difference however in the readability of R’s threshold maps and the default output is not easily interpreted.

## Conclusion

RUMM2030 provides a user friendly, complete approach to dichotomous and polytomous Rasch analysis, but is only accessible to researchers with sufficient funding to purchase a software dedicated to this one analysis. The statistical and graphical output can be interpreted and used immediately, it has significant documentation and support, and it has been widely used in Rasch publications. As R and the ltm, eRm, lordif and TAM packages are freely available, there are no financial barriers for access. Additionally, the open source nature of the underlying code base would allow for a third party to evaluate the statistical approach taken, and to assess the underlying reliability of the output. Two major advantages of R include that it can automate the entire process using a script and the portability of the R markup language to almost any other type of statistical techniques. RUMM2030 is a purpose-built software package specific to Rasch analysis whereas R is not-specific to Rasch analysis and can conduct almost any kind of statistical analysis. This approach is most accessible to those familiar with R as it does not require learning a new software package. For individuals naïve to either software package there will be a learning curve that will require the investment of time in order to be able to conduct Rasch analysis.

While the differences in output between RUIMM2030 and R can easily be explained by comparing the underlying statistical approaches taken by each program, there is disagreement on critical statistical decisions made by each program. This disagreement however should not be an issue as Rasch analysis requires users to apply their own subjective analysis, especially in the case of DIF, to ensure that it logical (i.e. DIF may be expected in some circumstances). While researchers might expect that Rasch performed on a large sample would be a stable, two authors who complete Rasch analysis of the PRWE found somewhat dissimilar findings [7, 11]. So, while some variations in results may be due to samples, this paper adds that some variation in findings may be software dependent. Both suggest that changing established measures based on a single Rasch analysis would be premature.

Limitations of this study include the reliance on 2 software packages, which cannot be generalized to other software that perform Rasch analysis. Further, we used built-in R functions whereas we could have used custom statistical analysis. PSI could have been evaluated by programming a custom function for PSI based on a known equation and the Thurstonian thresholds could have been converted into Rasch-Andrich thresholds for better comparison [13].

## Abbreviations

ANOVA: Analysis of variance
CTT: Classical Test Theory
DIF: Differential item functioning
ICC: Item characteristic curves
PCA: Principal component analysis
PRO: Patient-reported outcome measures
PSI: Person Separation Index

## References

[1] DeVellis RF (2006). Classical test theory. Med Care.

[2] Rasch G (1980). Probabilistic models for some intelligence and attainment tests.

[3] Mair P, Hatzinger R. Extended Rasch Modeling: The R Package eRm. PDF-Dateianhang zum Program eRm. 2009; Scheiblechner 1972. https://cran.r-project.org/web/packages/eRm/eRm.pdf.

[4] Rizopoulos D (2006). Ltm: an R package for latent variable modeling and item response theory analyses. J Stat Softw.

[5] Andrich D, Lyne A, Sheridan B, Luo G (2010). RUMM2030.

[6] Farzad M, MacDermid JC, Asgari A, Layeghi F. Validation of Persian version of patient rated wrist/ hand evaluation: confirmatory factor analysis and Rasch analysis. The University of Social Welfare and Rehabilitation Sciences Tehran, Iran; 2018.

[7] Esakki S, MacDermid J, Walton D, Grewal R, Packham T, Vincent J. Rasch analysis of the patient-rated wrist evaluation questionnaire. Arch Physiother. 2018;8:5. 10.1186/s40945-018-0046-z. 10.1186/s40945-018-0046-z PMC5828063 29497563

[8] Packham TL, MacDermid JC, Michlovitz SL, Buckley N. Content validation of the Patient-Reported Hamilton Inventory for Complex Regional Pain Syndrome: Validité de contenu du Hamilton Inventory for Complex Regional Pain Syndrome, une mesure des résultats déclarés par le patient. Can J Occup Ther. 2018;85(2):99–105. 10.1177/0008417417734562. Epub 2018 Feb 23. 10.1177/0008417417734562 29475370

[9] Tennant A, Conaghan PG (2007). The Rasch measurement model in rheumatology: what is it and why use it? When should it be applied, and what should one look for in a Rasch paper?. Arthritis Care Res.

[10] Pallant JF, Tennant A. An introduction to the Rasch measurement model: an example using the hospital anxiety and depression scale (HADS). Br J Clin Psychol 2007;46 Pt 1:1–18. doi:10.1348/014466506X96931. 10.1348/014466506x96931 17472198

[11] Packham T, Macdermid JC (2013). Measurement properties of the patient-rated wrist and hand evaluation: Rasch analysis of responses from a traumatic hand injury population. J Hand Ther.

[12] Fisher WP. Reliability statistics. Rasch Meas Trans. 1992;6:238.

[13] Bond T, Fox C (2001). Applying the Rasch model.

[14] Christensen KB, Kreiner S, Mesbah M. Rasch Models in Health. Hoboken: Wiley; 2013.

[15] Christensen KB, Olsbjerg M (2013). Marginal maximum likelihood estimation in polytomous Rasch models using SAS. Pub Inst Stat Univ Paris.

[16] Verhelst N (2001). Testing the unidimensionality assumption of the Rasch model. Methods Psychol Res Online.

[17] Spiel C, Gittler G, Sirsch U, Glück J. Application of the Rasch model for testing Piaget’s theory of cognitive development. Appl Latent Trait Latent Cl Model Soc Sci. 1997:111–7.

[18] Andrich D (1988). Rasch models for measurment.

[19] Andrich D, Luo G (2002). Conditional pairwise estimation in the Rasch model for ordered response categories using principal components. J Appl Meas.

[20] Linacre JM (2006). Demarcating category intervals. Rasch Meas Trans.

[21] Dorans N, Holland PW. DIF detection and description: mantel-haenszel and standardization. Educational Testing Service; 1991.

[22] Holland PW, Wainer H (1993). Differential item functioning.

[23] Andrich D, Lyne A, Sheridan B, Luo G. RUMM 2020. Perth; 2003.

[24] Christensen KB, Makransky G, Horton M (2017). Critical values for Yen’s Q3: identification of local dependence in the Rasch model using residual correlations. Appl Psychol Meas.

[25] Chambers JM (2009). Facets of R. R J.

[26] McGrath N, Dinn WM, Collins MW, Lovell MR, Elbin RJ, Kontos AP (2013). Post-exertion neurocognitive test failure among student-athletes following concussion. Brain Inj.

[27] What is R? R Foundation. https://www.r-project.org/about.html. Accessed 9 Mar 2017.

[28] MacDermid JC (2011). The patient-rated wrist evaluation (PRWE)© user manual. Hamilton, Ontario.

[29] MacDermid JC (1996). Development of a scale for patient rating of wrist pain and disability. J Hand Ther.

[30] Goldhahn J, Beaton D, Ladd A, Macdermid J, Hoang-Kim A (2013). Recommendation for measuring clinical outcome in distal radius fractures: a core. Ugeskr Laeger.

[31] Waljee JFJF, Ladd A, MacDermid JCJC, Rozental TDTD, Wolfe SWSW, Benson LS (2016). A unified approach to outcomes assessment for distal radius fractures. J Hand Surg Am.

[32] MacDermid JC, Turgeon T, Richards RS, Beadle M, Roth JH. Patient rating of wrist pain and disability: a reliable and valid measurement tool. J Orthop Trauma. 1998;12 http://journals.lww.com/jorthotrauma/Fulltext/1998/11000/Patient_Rating_of_Wrist_Pain_and_Disability__A.9.aspx. 10.1097/00005131-199811000-00009 9840793

[33] SP M, JC M, Richardson J, NJ M, Grewal R (2015). A systematic review of the measurement properties of the patient-rated wrist. J Neurosurg Spine.

[34] Rizopoulus D. Latent Trait Models under IRT. CRAN. 2015; https://cran.r-project.org/web/packages/ltm/ltm.pdf.

[35] Mair P, Hatzinger R, Maier M (2016). Package ‘ eRm .’ CRAN.

[36] Streiner D, Norman G. Health measurement scales: a practical guide to their development and use. 3rd edition: Oxford University Press; 2003.

[37] Hagquist C, Bruce M, Gustavsson JP (2009). Using the Rasch model in nursing research: an introduction and illustrative example. Int J Nurs Stud.

[38] Smith EV Jr. Understanding Rasch measurement: detecting and evaluating the impact of multidimenstionality using item fit statistics and principal component analysis of residuals. J Appl Meas. 2002. 12011501

[39] Tennant A, Pallant JF (2006). Unidimensionality matters. Rasch Meas Trans.

[40] Robitzsch T, Wu A, Robitzsch M. Package ‘TAM.’ CRAN; 2016. https://cran.r-project.org/web/packages/TAM/index.html.

[41] Choi SW, Gibbons LE, Crane PK (2011). Lordif : an R Package for detecting differential item functioning using iterative hybrid ordinal logistic regression/item response theory and Monte Carlo simulations. J Stat Softw.

